# Rwanda – lasting imprints of a genocide: trauma, mental health and psychosocial conditions in survivors, former prisoners and their children

**DOI:** 10.1186/1752-1505-7-6

**Published:** 2013-03-26

**Authors:** Heide Rieder, Thomas Elbert

**Affiliations:** 1 Department of Psychology, University of Konstanz, Konstanz, Germany

**Keywords:** Rwanda, PTSD, Anxiety, Depression, Risk factors, Genocide survivors, Former prisoners, Descendants

## Abstract

**Background:**

The 1994 genocide of the Tutsi in Rwanda left about one million people dead in a period of only three months. The present study aimed to examine the level of trauma exposure, psychopathology, and risk factors for posttraumatic stress disorder (PTSD) in survivors and former prisoners accused of participation in the genocide as well as in their respective descendants.

**Methods:**

A community-based survey was conducted in four sectors of the Muhanga district in the Southern Province of Rwanda from May to July 2010. Genocide survivors (*n* = 90), former prisoners (*n* = 83) and their respective descendants were interviewed by trained local psychologists. The PTSD Symptom Scale Interview (PSS-I) was used to assess PTSD, the Hopkins Symptom Checklist (HSCL-25) to assess symptoms of depression and anxiety and the relevant section of the M.I.N.I. to assess the risk for suicidality.

**Results:**

Survivors reported that they had experienced on average twelve different traumatic event types in comparison to ten different types of traumatic stressors in the group of former prisoners. According to the PSS-I, the worst events reported by survivors were mainly linked to witnessing violence throughout the period of the genocide, whereas former prisoners emphasized being physically attacked, referring to their time spent in refugee camps or to their imprisonment. In the parent generation, when compared to former prisoners, survivors indicated being more affected by depressive symptoms (*M* = 20.7 (SD = 7.8) versus *M* = 19.0 (SD = 6.4), *U* = 2993, *p* < .05) and anxiety symptoms (*M* = 17.2 (SD = 7.6) versus *M* = 15.4 (SD = 7.8), *U* = 2951, *p* < .05) but not with regard to the PTSD diagnosis (25% versus 22%, *χ*^*2*^(1,171) = .182, *p* = .669).

A regression analysis of the data of the parent generation revealed that the exposure to traumatic stressors, the level of physical illness and the level of social integration were predictors for the symptom severity of PTSD, whereas economic status, age and gender were not. Descendants of genocide survivors presented with more symptoms than descendants of former prisoners with regard to all assessed mental disorders.

**Conclusions:**

Our study demonstrated particular long-term consequences of massive organized violence, such as war and genocide, on mental health and psychosocial conditions. Differences between families of survivors and families of former prisoners accused for participation in the Rwandan genocide are reflected in the mental health of the next generation.

## Introduction

In April 1994, Rwanda was immersed in a brutal wave of organized violence that left an estimated one million people dead in a period of only three months. Civil war, genocide against the Tutsi minority group and violent reprisal attacks until 1998 horrified its inhabitants. The thoroughly planned and state-monitored genocidal violence was specifically marked by the extensive participation of the local population: neighbors went after neighbors by means of guns, machetes or sticks during house to house searches, at roadblocks or at central congregation points. Looting, destroying property and genocidal acts including murder and sexual violence were common [1]. Overall, more than 10% of the country’s 7.8 million population and approximately 75% of the Tutsi ethnic minority were killed and a huge number of people ended up widowed or orphaned. In the direct aftermath of genocide, two million people took refuge in the neighboring countries. Many of them did not reenter Rwanda prior to 1996, when the refugee camps began breaking down and people felt encouraged and/or coerced to return. In many cases, a return to Rwanda was followed by immediate incarceration. The release of these prisoners did not begin before 2002 when *Gacaca*, a judicial initiative based on a traditional Rwandan mechanism of local conflict resolution, was implemented to confront the estimated 1.2 million cases [2]. Until the present day, genocide survivors and those who participated in the genocide continue to live next door to each other.

In the aftermath of 1994, genocide survivors showed high rates of mental health and psychosocial problems due to the inconceivable, dehumanized brutality that the majority of them had been exposed or witness to. Entire family systems as well as the general social fabric that formerly provided support were destroyed due to losses of family members and growing mistrust and fear following the genocide. A great majority of the survivors were female and woman-headed households proved to be especially vulnerable, suffering from the effects of economic deprivation, which included a lack of food, housing and money for the education of their children [3]. Apart from general population samples, studies analyzing the mental health situation in Rwanda following the genocide have mainly focused on groups of widows and orphans or children living in child-headed households. An elevated level of depressive and anxious symptoms as well as PTSD was found in each of these groups [4-7]. On the other hand, little to nothing is known about the mental health situation of former prisoners in Rwanda, many of which spent several years in refugee camps after 1994. It is assumed that former prisoners – that is, accused perpetrators and their respective families – also present with mental health problems, whether due to their participation in [8,9] or exposure to violence, genocide and their refugee status. Concrete data on this is, however, currently lacking.

To our knowledge, no existing study has used data from a local population sample investigating both survivors and former prisoners as well as their respective descendants living together in the same communities. We opted for a comparison of these two, as they constitute important stakeholders within the post-conflict Rwandan society and assumingly present with different mental health and socioeconomic profiles. This also assumed that a neutral, unaffected group would be near impossible to find owing to the pervasive effects of violence on the Rwandan population as a whole. As the primary targets of genocidal acts were men and boys, genocide survivors proved to be mainly women, whereas genocide-related crimes were mainly committed by men [10]. A gender imbalance was therefore assumed in the examined sample of parents. We furthermore hypothesized that families of genocide survivors had been exposed to more traumatic events and that they were more affected in terms of mental and physical health. With regard to social and economic factors, we assumed that they live under worse conditions than the group of former prisoners and their descendants.

## Methods

### Sampling and procedure

The inclusion criterion for all participants born prior to 1994 was that they must have resided in Rwanda in the same year, while parents of children born prior to 1994 had to be at least 18 years old at the time of genocide. In the present study, survivors were defined as targets of the 1994 genocide and therefore represent today’s *rescapés*. They were mainly categorized as Tutsi but were in some cases Hutu as well (e.g., Hutu women who were persecuted for being married to a Tutsi). Both groups were thus included in the survey. Former prisoners were defined as released prisoners who in the aftermath of genocide were incarcerated because of being suspected of participation in genocide. Therefore, all former *génocidaires* were included, even if they claimed to be innocent, declaring that they had not killed or harmed anyone. Following this definition, we were interested in the local perception of the family as a “perpetrator family” rather than in the legal status of the individuals.

The survivor sample consisted of 64 women (71.1%) and 26 men (28.9%) and the former prisoner sample consisted of eight women (9.8%) and 74 men (90.2%). In the group of descendants, eligible participants had to be between 19 and 31 for those born prior to the genocide and between 13 and 15 for those born after 1994. The sample of descendants of survivors consisted of 55 women (56.7%) and 42 men (43.3%) and the sample of descendants of former prisoners consisted of 45 women (49.5%) and 46 men (50.5%). Eight participants of the 368 refused to participate in the study for reasons of mistrust, lack of further financial support and owing to the fear of being sent back to prison.

The present study was designed as a cross-sectional survey of a local population sample in the Muhanga district of the Southern province of Rwanda. Muhanga and its main provincial town Gitarama are located in the geographic center of the country, one hour away from the capital of Kigali. Before 1994, the province’s population was characterized by cohabitation and a high incidence of intermarriage between Hutu and Tutsi [11,12]. By choosing this research site, we intended to investigate communities where both former ethnic groups (and to a small amount Twa, the third population group) live alongside one another until today and that have an elevated number of Tutsi in contrast, for instance, to districts in the North. Four sectors were selected within the Muhanga district (Nyamabuye, Muhanga, Shyogwe, Cyeza) using a simple random sampling approach. Approval for the implementation of the survey was obtained from the National Institute of Statistics of Rwanda, Kigali (NISR), and from the Ethical Review Board of the University of Konstanz. Furthermore, district and sector representatives were informed of the survey and provided a research permit in the local language of Kinyarwanda. All participants provided their written informed consent and parents signed for their children after having received extensive information on the aim of the study. Interviews were conducted by seven local Bachelor-level psychologists (four men and three women) who had participated in former epidemiological studies and had thus already received extensive training and experience in conducting structured interviews. An additional week of training was provided to all interviewers in order to review, and afterwards practice unfamiliar parts of the interview by means of role-playing and group discussions. Interviewers were closely supervised throughout the entire procedure by a female clinical psychologist (H.R.). Whenever needed, follow-up care of the interviewees was provided. If necessary, participants– especially victims of sexual violence – were referred to self-help groups or trauma counselors for further care.

The study was conceived as a community-based study with a house-to-house survey. In all four selected sectors, two quarters were randomly chosen (one quarter per interviewer) and interviewers went door-to-door, starting at a convenient location within the assigned sector. Each subsequent house was approached until the required number of interviews was achieved. The first adult person encountered in the household was asked whether genocide survivors or former prisoners resided in the house. As soon as it was determined that one eligible person and his or her child met the conclusion criteria, the interviewer started the interview with the person already spoken with. When two or more eligible adult persons were present, one of these was randomly selected for participation. If no one was at home at the time of the first visit, interviewers returned afterwards to ask for eligible participants. If eligible parents had children that met criteria for both age ranges (born before or after the genocide), both children were interviewed. If more than one child within one age group was available and willing to participate, one of these was randomly chosen. Each interviewee received 1,000 Rwandan Francs (about 1.30 Euro) as compensation for the time spent in the interview. The interviews lasted between one and two hours, were carried out individually in the respondent’s home and took place between May and July 2010.

### Measures

The socio-demographic part of the interview contained questions about gender, age, marital status, educational background as well as some economic and social variables. A social integration index was built by integrating the number of current close friends, the participation in any community activity and the integration in cooperatives or associations. The economic index contained the following variables: possessions (house, agricultural fields), any monthly monetary income, the capacity to satisfy the family’s needs and facts on typical nutrition (number of meals, with protein or not). Both the social integration and economic indices were calculated by the addition of their z-transformed variables divided by the square root of their number. These indices were only calculated for the parent generation, as these variables were not assessed for descendants born after 1994 and could therefore not be calculated for the whole group of descendants. Furthermore, six questions on physical health within the previous six months were included, referring to common symptoms or syndromes in Rwanda (e.g., chronic pain or diarrhea, tuberculosis, HIV or any kind of disability). Answers were compiled on an index of physical illness with a possible range of 0 to 6. Finally, questions concerning the experience of displacement and loss due to war and genocide and with regard to the group of former prisoners, their time spent in prison, were added.

To assess trauma confrontation, the Rwandan adjusted Event Scale was used [5]. This contained 25 potentially traumatic events, for example, *to witness a massacre, to be physically attacked* or *to hide under cadavers*. Each type of event was assessed referring to a specific period of time, namely *before, during* and *after* the genocide, as well as *lifetime* exposure. In all four categories, events were summed up to a total number of event types to which participants had been exposed.

Diagnostic status and symptom severity of PTSD were determined using the PTSD Symptom Scale Interview (PSS-I) [13], which assesses the 17 symptom criteria in the previous month according to the *DSM-IV*. Each item was evaluated on a four-point Likert scale ranging from 0 (not at all/only one time) to 3 (five or more times a week/almost always) and a PTSD severity score with a possible score range of 0 to 51 was established for every subject by adding all symptom scores. The Kinyarwandan version of this was first produced by colleagues conducting research in the Ugandan refugee camp Nakivale and was later also applied in different settings in Rwanda [9,14]. Its translated version demonstrated satisfactory psychometric properties [15,16].

Depressive and anxiety symptoms in the week prior to the interview were administered by the use of the Hopkins Symptom Checklist (HSCL-25) [17]. For further analysis, a severity score for anxiety (scores range from 10 to 40) was established and syndromal anxiety was estimated by using a critical cut-off value for clinical relevance at a mean score of 1.75 [18,19]. A symptom score for depression (scores range from 15–60) was established and again, syndromal depression was estimated using a critical cut-off value of 1.75. For further analysis of the validity of this procedure, a cross-check was made by also establishing syndromal depression according to the *DSM-IV* criteria using an algorithm suggested by Bolton and colleagues [20]. At least five out of nine depressive symptoms including at least one of the depressed mood items (e.g., crying easily, feeling hopeless) had to be present to fulfill the diagnosis, whereas functional impairment was not further assessed. The risk of suicidality was estimated by means of the corresponding section of the Mini International Neuropsychiatric Interview (M.I.N.I.) [21] and two items about alcohol and other drug abuse in the previous week were added.

All instruments were applied in Kinyarwanda language versions, produced by colleagues and applied before in Rwanda. All diagnostic scales (including the self-rating scales) were applied in the form of a clinical interview.

### Statistical analyses

Descriptive data are presented as frequencies (%), mean scores and standard deviations. Chi square analysis and Mann–Whitney tests are used to analyze between-group differences. To explore the impact of different predictor variables on the severity of the posttraumatic stress symptom score in the group of adult survivors, a regression analysis was calculated. As the PTSD symptom score is a count variable and as our data did not fulfill the assumptions to run a linear regression, we applied an extended generalized linear model for count data based on the assumption of a negative binomial distribution of the data. The Lagrange multiplier test statistic revealed that overdispersion of the data was not a problem (*χ*^*2*^(1,176) = 3.8, *p* = .052); a Poisson regression was thereby performed. The predictor variables included in the model were age, gender, exposure to traumatic stressors (number of traumatic events), physical illness, the social integration index and the economic index. Spearman’s Rho correlations are used to further investigate links between different socio-demographic variables. Data analysis was conducted using SPSS software version 20.

## Results

For further discussion, it must first be considered that socio-demographic profiles between groups differed significantly in that survivors were mainly female (71%), more educated and half of them were widowed (53%), whereas former prisoners were mainly male (90%), presented fewer years of schooling and were significantly more often married (83%). A detailed description of the characteristics of the sample can be drawn from Table 1.

**Table 1:** Sample characteristics

	**Survivors(*n*** **= 90)**	**Former prisoners(*n*** **= 83)**	***Statistics***	**Descendants of survivors(*n*** **= 97)**	**Descendants of former prisoners(*n*** **= 90)**	***Statistics***
Gender % *(n)*						
Male	28.9 (26)	90.2 (74)	*χ*^*2*^(1,172) = 66.4***	43.3 (42)	50.5 (46)	n.s.
Female	71.1 (64)	9.8 (8)		56.7 (55)	49.5 (45)	
Age	51.6 (10.3, 30–77)	53.9 (7.9, 34–81)	n.s.	21.1 (5.9, 13–31)	21.6 (5.8, 13–31)	
Marital Status % *(n)*						
Single	4.5 (4)	1.2 (1)	n.s.	91.8 (89)	85.7 (78)	n.s.
Married	38.9 (35)	82.9 (68)	*χ*^*2*^(3,172) = 34.6***	6.2 (6)	13.2 (12)	n.s.
Divorced/ Separated	3.3 (3)	4.9 (4)	n.s.	2.0 (2)	1.1 (1)	n.s.
Widowed	53.3 (48)	11.0 (9)	*χ*^*2*^(3,172) = 34.7***	-	-	
Number of children *M (SD, range)*	6.0 (2.76, 1–13)	6.8 (2.63, 2–16)	*U* = 2971.5*	0.3 (1.03, 0–5)	0.3 (0.92, 0–6)	n.s.
Years of Schooling *M (SD, range)*	5.2 (3.48, 0–14)	3.7 (3.28, 0–14)	*U* = 2811.5**	6.71 (3.18, 0–16)	5.42 (3.02, 0–17)	*U* = 3251.0**
Highest degree obtained % *(n)*						
No degree	48.9 (44)	61.0 (50)	n.s.	39.2 (38)	58.2 (53)	*χ*^*2*^(2,188) = 6.8**
Primary School	46.7 (42)	35.4 (29)	n.s.	45.4 (44)	34.1 (31)	n.s.
Secondary School	4.4 (4)	3.6 (3)	n.s.	15.4 (15)	7.7 (7)	n.s.
Possessions % *(n)*						
House	93.3 (84)	98.8 (81)	n.s.	n.a.	n.a.	
Agricultural Field	80.0 (72)	96.3 (79)	*χ*^*2*^(1,172) = 10.7***	n.a.	n.a.	
Monthly Income *M (SD, range)*	2922.2 (6849.5, 0–41.000)	3695.1 (6051.6, 0–35.000)	n.s.	n.a.	n.a.	
Social Integration						
Number of friends *M (SD, range)*	4.3 (4.0, 0–20)	4.9 (3.7, 0–20)	n.s.	4.3 (4.5, 0–25)	4.6 (3.6, 0–20)	n.s.
Community activities % *(n)*	94.4 (85)	92.7 (76)	n.s.	n.a	n.a.	
Memberships in cooperatives *M (SD, range)*	.7 (.8, 0–3)	.7 (.9, 0–5)	n.s.	n.a.	n.a.	
Family members lost in 1994 *M (SD, range)*	14 (1.6, 0–70)	3 (.4, 0–24)	*U* = 1032.5***	n.a.	n.a.	
Displacement due to genocide % *(n)*	98.9 (89)	82.9 (68)	*U* = 13.7***	n.a.	n.a.	
Destination of displacement % *(n)*				n.a.	n.a.	
Same district	75.5 (68)	15.9 (13)	*χ*^*2*^(1,172) = 61.4***	n.a.	n.a.	
Other district (Bugesera, Kibuye)	23.3 (21)	59.8 (49)	*χ*^*2*^(1,172) = 23.6***	n.a.	n.a.	
Foreign country (DRC)	-	7.3 (6)	*χ*^*2*^(1,172) = 6.8**	n.a.	n.a.	
Years spent in prison *M (SD, range)*	n.a.	8.5 (4.4, .2-15.7)		n.a.	n.a.	

Note: *p < .05; **p < .01; ***p < .001.

n.a. = not assessed as data for the generation born after 1994 was missing.

### Exposure to war and genocide-related events

**Figure 1:**
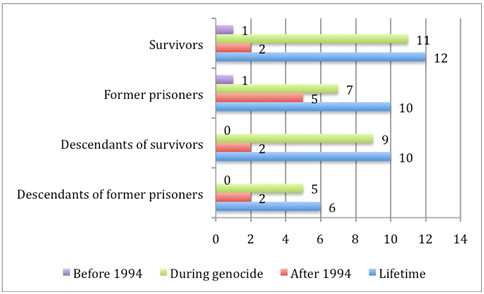
**Number of traumatic event types (*Mean*) before 1994, during genocide and after 1994, and in lifetime reported by respondents born before 1994.**

Respondents reported having been exposed to about eight different types of traumatic stressors. The average number of events experienced by each group with regard to different periods of time is illustrated in Figure 1. No gender difference was found in the parent generation with regard to the number of events ever experienced, but women reported a higher trauma load with regard to the period of the genocide (*U* = 2446.0, *p* = .0001) and men reported a higher trauma load with regard to the aftermath of the genocide (*U* = 1880.0, *p* = .0001). In the group of descendants, no gender difference was found at all. Potentially traumatic events such as *being captured or kidnapped, witnessing a massacre, serious injury* or *attack with a weapon* as well as *sexual abuse or rape* were mainly reported by the survivor group and their descendants and linked to genocidal violence. *Confrontation with other war or combat situations* linked to political events in 1959 or 1973 as well as to the war against infiltrators from 1996 to 1998 were reported by the parent generation of the survivor group (43% and 20%) and to a lesser extent by former prisoners (18% and 9%). On the other hand, 65% of the latter group reported having experienced *physical attack* especially after the genocide and thereby mainly in relation to their imprisonment as well as their time spent in camps for Internally Displaced Persons (IDP) in Kibuye in the Western region of Rwanda or in refugee camps in the Democratic Republic of Congo (DRC).

**Figure 2:**
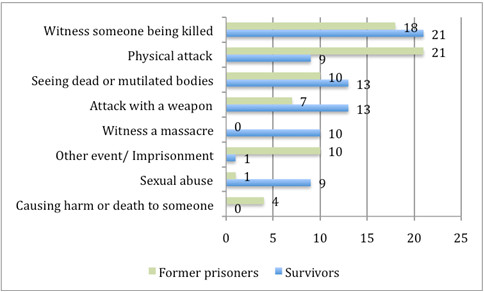
**Percentages of the worst traumatic event types reported by the group of survivors (*n*** **= 90) and former prisoners (*n*** **= 83).**

**Figure 3:**
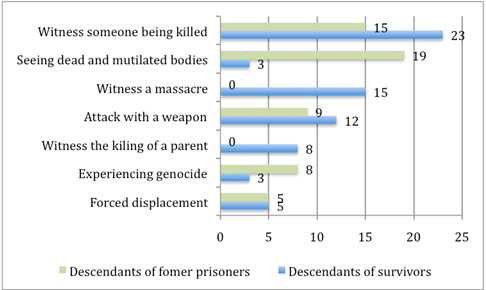
**Percentages of the worst traumatic event types reported by the group of descendants of survivors (*n*** **= 66) and descendants of former prisoners (*n*** **= 62), all born before 1994.**

*Witnessing the killing of someone*, *seeing dead and mutilated bodies*, *being attacked with a weapon* and *physical attack* proved to be the most upsetting experiences reported by the entire group. However, survivors and former prisoners as well as their descendants reported different types of events to be the worst (see Figures 2 and 3). Whereas former prisoners reported being mostly affected by their own experience of being *physically attacked* and incarcerated, the three other groups mentioned mainly *witnessing the killing of someone* to be worst.

### Level of distress

The analysis of the PDS data revealed that 81% of the whole sample fulfilled the A1 criterion for a traumatic event according to the *DSM-IV*. The prevalence rate for current PTSD was 25% in the survivor group, 16% in all their descendants and 22.4% in their descendants born before 1994. 24.6% of all widows and widowers fulfilled this diagnosis. 22% of the group of former prisoners, 1% of all their respective descendants and 1.6% of their descendants born before 1994 suffered from PTSD. The severity score of the PDS ranged from 8 to 48 (*M* = 23.9, *SD* = 10.3) for those who fulfilled the diagnosis. In the parent generation, intrusions associated with the B cluster of the PTSD diagnosis turned out to be as frequent in survivors as in former prisoners. The HSCL severity score for anxiety ranged from 10 to 40 (*M* = 14.6, *SD* = 6.7). 37% of the survivor group and 23% of their descendants were above the cut-off value for clinical relevance. In families of former prisoners, 22% of the parent generation and 8% of their descendants were thus likely to suffer from an anxiety disorder. The HSCL severity score for depression ranged from 15 to 55 (*M =* 18.2, *SD* = 5.9). Considering a cut-off score, 21% of the survivors and 3% of their descendants as well as 14% of the former prisoners and 5% of their respective descendants were likely to suffer from depression. Following the Bolton algorithm as earlier described, 30% of the survivor group and 16% of their descendants might have been ill with symptoms of an affective disorder and 15% of the former prisoners as well as 5% of their respective descendants likewise. Prevalence rates resulting on the one hand from a cut-off mean score of 1.75 and on the other hand from symptom criteria of the *DSM-IV* diagnosis for major depression thereby proved not to be in accordance with each other. The analysis of the M.I.N.I. data revealed that 11% of the sample reported suicidal tendencies. One quarter of the survivor group and 7% of the former prisoners turned out to be at risk. 15% of all former prisoners reported alcohol abuse in the week prior to the interview, whereas only 7% of victims reported this. In the parent generation, women showed a higher level of suicidality than men (*U* = 2826.0, *p* = .000) and a lower level of alcohol abuse (*U* = 3120.0, *p* = .005). All group differences in psychopathology are presented in Table 2.

**Table 2:** Psychopathology in survivors and former prisoners of the Rwandan genocide and their respective descendants

**Measures**	**Survivors**	**Former prisoners**	***Statistics***	**Desc. of survivors**	**Desc. of former prisoners**	***Statistics***
PTSD						
Diagnosis % *(n)*	24.7 (22)	22.0 (18)	n.s.	15.5 (15)	1.1 (1)	*χ*^*2*^(1,188) = 12.4***
Severity score *M (SD)*	10.2 (8.1)	11.1 (10.8)	*U* = 2753.0*	5.3. (8.3)	1.4 (3.8)	*U* = 2866.5***
- Cluster: Intrusions	3.9 (4.0)	3.9 (4.4)	n.s.	2.0 (2.9)	.8 (1.8)	*U* = 3022.0***
- Cluster: Avoidance	3.9 (4.6)	2.9 (4.3)	*U* = 2846.5*	1.8 (3.2)	.6 (1.8)	*U* = 3271.5***
- Cluster: Arousal	2.8 (3.6)	1.9 (3.2)	*U* = 2797.0**	1.4 (2.8)	.2 (1.0)	*U* = 3228.5***
Anxiety						
Severity score *M (SD)*	17.2 (7.6)	15.4 (7.8)	*U* = 2951.0*	13.9 (5.5)	12.2 (4.7)	*U* = 3445.0**
Depression						
Severity score *M (SD)*	20.7 (7.8)	19.0 (6.4)	*U* = 2993.0*	17.1 (4.1)	16.1 (3.0)	*U* = 3759.0*
Alcohol Abuse % *(n)*	6.7 (6)	14.6 (12)	n.s.	2.1 (2)	3.3 (3)	n.s.
Drug Abuse % *(n)*	2.2 (2)	1.2 (1)	n.s.	1.0 (1)	1.1 (1)	n.s.
Suicidality % *(n)*						
None	75.3 (67)	92.7 (76)	*χ*^*2*^(1,172) = 9.4**	89.7 (87)	97.8 (89)	*χ*^*2*^(1,188) = 5.1*
Low level	18.0 (16)	4.9 (4)	*χ*^*2*^(1,172) = 7.1**	7.2 (7)	2.2 (2)	n.s..
Medium level	2.3 (2)	-	n.s.	1.0 (1	-	n.s.
High level	4.5 (4)	2.4 (2)	n.s.	2.1 (2)	-	n.s.

Note: *p < .05; **p < .01; *** *p <* .001.

### Association between PTSD symptom severity and related disorders

Positive correlations were found between the PDS symptom severity score and other related disorders in the total sample *(N* = 360). The symptom severity score of PTSD was associated with the severity of depressive symptoms (Spearman, *r* = .62, *p* = .000) and symptoms of anxiety (*r* = .59, *p* = .000). Depression and anxiety symptom scores were associated as well (*r* = .69, *p* = .000). PTSD symptom severity occurred to be linked to suicidal tendencies (*r* = .35, *p* = .000), but neither alcohol nor other drug abuse.

### Physical health

In the parent generation, general complaints such as headaches, coughing and malaria (51.2%) were most frequently reported, followed by chronic pain (32.0%) and HIV (7.0%) especially amongst survivors as well as disability due to genocide-related violence (5.8%). Survivors were more affected and differed significantly from the group of former prisoners in the physical illness index (*U* = 2896.0, *p* = .009). Again, a gender difference was found as women reported more physical symptoms (*U* = 2640.0, *p* = .001). Descendants of survivors seemed as well to be more affected than descendants of former prisoners, while this difference did not reach significance (*U* = 3742.0, *p* = .058). Among those, general complaints (44.2%) especially about stomachaches as well as chronic pain (5%) were again reported most frequently.

### Prediction of traumatic stress in the parent generation

Predictors of traumatic stress were analyzed by calculating a negative binomial regression on the PDS symptom severity score of the parent generation (see Table 3). Poor physical health, a low level of social integration as well as a high exposure to war and genocide were the strongest predictors for PTSD symptom severity, whereas age, gender and economic status were not.

**Table 3:** **Prediction of PTSD symptoms in the parent generation (*n*** **= 176) resulting from a negative binomial regression analysis**

**Predictor Variables**	**Beta**	**SE**	***p*-value**
Age	-.01	.01	.677
Gender (Male)	-.35	.20	.073
Physical Illness	.32	.12	.010
Economic Status	.05	.03	.065
Social Integration	-.17	.08	.026
Exposure to war and genocide (Number of event types)	.17	.03	.000

Note: *χ*^*2*^ (6,176) = 67.1, *p* < .001.

### Socio-economic conditions in the parent generation

Regarding the two indices, the social integration and the economic index, the parent generation did not differ significantly in the first, but rather in the latter one (*U* = 2699, *p* = .006) identifying the group of former prisoners as the wealthier one. While further analyzing gender differences, men presented a higher level in both the economic (*U* = .2723.0, *p* = .006) and the social indices (*U* = 2805.0, *p* = .029). And, with regard to marital status, widows and widowers showed a lower economic status (*U* = 2054.0, *p* = .000), but no difference in the social integration index compared to non-widowed. A negative correlation between physical illness and both indices, the social integration (*r* = −.20, *p* = .000) and economic status (*r* = −.33, *p* = .000) was found.

## Discussion

The present study examined mental health problems and psychosocial conditions in Rwandan families 16 years following the 1994 genocide. Its main aim was to investigate the impact of war, genocide and other potentially traumatic experiences on genocide survivors on the one hand and former prisoners on the other hand, as well as the respective descendants of both groups. The study also examined correlates of PTSD and the prediction of the PTSD symptom severity. In general, survivors and their descendants reported more traumatic events and proved to be more affected than families of former prisoners. Posttraumatic stress reactions were especially elevated in adult survivors who had experienced a high number of traumatic events, had poor physical health and were lacking in social integration.

Not surprisingly, survivors and their descendants, as the primary targets of the 1994 atrocities, showed the highest exposure to traumatic stressors with twelve and ten different event types, thus reflecting their exposure to genocide-related violence. These findings are in line with other studies conducted in Rwanda [4,5,9]. The average number of event types reported by former prisoners and their descendants ranged from six to nine events and was also mainly linked to the period of genocide, although both generations in this group emphasized its aftermath more frequently than the families of survivors. Former prisoners especially pointed to physical attacks experienced in refugee camps in the eastern Congo or related to the imprisonment upon their return. Their descendants often became witnesses of these imprisonments and the circumstances under which they took place. In this way, the past and recent political situations in Rwanda, which were marked by various episodes of persecution, attack, massacre, and forced displacement, were also directly reflected in the number of events reported before and after 1994. This furthermore highlighted the repetitive and cumulative nature of trauma in Rwanda and the Great Lakes Region, which is not only limited to genocide.

In the present study, 25% of the genocide survivors and 22% of the former prisoners were diagnosed with PTSD. In Rwanda, studies reported 25%-29% of PTSD in non-specified adult populations [6,22,23], 41%-51% in widows and genocide survivors [6,7] and 37% within the Southern province of Rwanda [24]. Our sample therefore showed a lower level of distress than previously reported data on Rwanda, while also presenting a high trauma load. In addition, it is in discordance with data collected in the Southern province. These differences might be due to recovery over time [25] but might also be linked to differences in exposure to genocide within the same province. As stated by Straus [26], Gitarama manifested less “anti-Tutsi violence” in comparison to other Southern cities such as Butare or Gikongoro, and, according to des Forges [27], the nearby Kabgayi church offered special protection to a great number of Tutsi in the area. While in the present study genocide survivors and former prisoners significantly differed in their PTSD severity scores, this was not the case with syndromal PTSD. This was due to the fact that both groups manifested the same level of intrusions – the B criterion of the *DSM-IV* diagnosis. While this elevated level of intrusions seems unsurprising in genocide survivors, it needs further explanation with regard to former prisoners. The prisoners examined in the present study had spent about eight years in prison: some of them were incarcerated in the direct aftermath of the genocide and others upon their return from refugee camps, as only a few did not leave their home district in 1994. For those prisoners, to be put in prison might have felt like the point of no return as the accused did not necessarily expect to ever leave prison or at least not until the implementation of *Gacaca* jurisdiction in 2002. Adverse experiences throughout their prison time such as malnutrition, lack of appropriate health care, overcrowded detention conditions or physical harassment and attacks might have added new traumatic events to the already existing fear network [28], and feelings of hopelessness and helplessness over the years might have fostered and maintained intrusive symptomatology. Acute fear, one might argue, characterized their time in prison as well as the arrival in their respective communities as they did not know what to expect and how they would be perceived by others in this changed political environment.

Comparing rates of symptoms of anxiety and depression between survivors and former prisoners, the former showed a significantly higher level of distress. With regard to the HSCL score, 37% of survivors and 22% of former prisoners fulfilled the criteria for anxiety disorder, and according to the Bolton algorithm 30% versus 15% for depression. When relying on these results, our findings are consistent with earlier reported studies on survivors and widows or on the general Rwandan population [6,7,20,23,29], even though no comparable data are available for former prisoners. A high level of disagreement was nonetheless found when comparing prevalence rates gained either by using a cut-off score or following *DSM-IV* symptom criteria, as suggested by Bolton and others [20], to screen for depression. In a recently published study, Ertl et al. [16] critically discussed the unevaluated adjustment of a cut-off score developed in a different context – that is, for example, not appropriate to the given East African situation. Therefore, in future research, the HSCL might be better applied to investigate symptom severity instead of prevalence rates based on a specific cut-off value.

Suicidal tendencies were found in 25% of all survivors and in 7% of the group of former prisoners, occurring more often among women. Rates showed to be lower compared with an earlier reported study [7], but still displayed a considerable level of distress in a society where suicidal tendencies had not previously been commonly reported and were rejected by the majority of our sample as, to quote a participant, “an inappropriate way to solve problems for any Rwandan believing in God”. Alcohol consumption occurred twice as much in the group of former prisoners (15%) than in the survivors and was especially linked to males. This had not received much attention in earlier reported studies on Rwanda and is not easily admitted to by Rwandans, whose sense of disclosure prohibits openly talking about sensitive topics, even while the consumption of locally produced alcohol is a common phenomenon in rural areas and symbolic for good neighborhood relationships [30]. To further differentiate between general alcohol consumption and clinically relevant problems associated to alcohol and other substances, a recent representative study by the Rwandan Ministry of Health [23] examined a general population sample and found rates of drug and alcohol abuse ranging from 3%-6% and alcohol addiction of 5%-7%. Even while our data do not allow for any causal attribution, one might argue that these two specific features, suicidality and alcohol abuse, point to possible reactions and mechanisms for dealing with loss and trauma. Family dynamics might therefore be affected by these issues, especially if the broader family and community support is broken. Furthermore, it is possible that the experience of war and violence can lead to an elevated level of family violence, which often turns out to be moderated by alcohol abuse in a parent. Catani et al. [31] demonstrated an association between the father’s alcohol intake and maltreatment reported by his children in a Sri Lankan sample of children affected by long-lasting conflict. Although systematically collected data on domestic violence in Rwanda are scarce, another recent study reported that alcoholism ranges under the first three causes of aggressive and violent behavior towards intimate partners or children in this country [32]. Therefore, psychological disorders within the local population following experiences such as war and genocide, including alcohol and drug abuse, need to be considered in community-based interventions, which tend to diminish the risk of further violence on following generations.

Overall, the present study demonstrated a high degree of co-morbidity between diverse disorders, as postulated earlier [33-35]. Altogether, one quarter of all adult survivors suffered from PTSD, clinically relevant depression and/or anxiety, reflecting the serious mental health situation as well as the long-term consequences of massive violence even 16 years following the genocide [36-38].

With regard to the group of descendants, our study revealed that 16% of the descendants of survivors compared to only 1% of the descendants of former prisoners (and none of those born after 1994) fulfilled the *DSM-IV* criteria for the diagnosis of PTSD. In a study on a general sample of Rwandan youth interviewed during the direct aftermath of the genocide, Neugebauer et al. [39] reported a PTSD rate of 62%. Recent research on vulnerable groups such as orphans showed lower PTSD rates, between 24% and 34% [5,7,40]. With regard to those born before 1994, our sample of descendants of survivors manifested a similar level of PTSD to those reported by these last studies. Among these descendants, a particularly high trauma load was found and 50% showed to be half-orphaned. Their specifically vulnerable and life-threatening situation in 1994 and afterwards was strongly shaped by their families’ experiences. Due to persecution and death, parental protection throughout the period of violence was often missing. In the aftermath of the genocide, their families had to cope with severe circumstances and descendants often took over great responsibilities, which often continue today and might explain the ongoing sequelae of distress as depressive and anxiety symptoms [7]. The group of descendants of former prisoners within the present sample, however, differed even more from the youth described in Neugebauer’s study, though concrete comparable data is missing. Throughout the genocide, descendants of former prisoners did not necessarily flee with their families, but rather stayed at home or were individually sent to other remaining family members. When they had to take refuge with their families who were moving to the western parts of Rwanda, they often went in groups and the mainly Hutu background of their mothers offered them special protection in comparison to the descendants of survivors. Even if they had witnessed war and genocidal violence, they had never been specifically targeted, as the primary aim of the genocide perpetrators was to eliminate the group of Tutsi and their families [24]. Finally, information on what was going on in Rwanda in 1994 was scarce. A lack of cognitive understanding of the dimension of the events might therefore also have modulated the affect regulation and in turn have added a protective factor for those children [41]. Our data furthermore suggest that younger children born after 1994 did not specifically suffer from PTSD or other mental disorders. Further research is needed to better understand potential transgenerational effects of genocide on those children who did not live through the genocide in comparison to their older siblings [42]. Apart from these family issues, when referring to previous studies the broader social climate is in question as well, thereby demonstrating a clear association between mental health problems such as PTSD and feelings of hatred and revenge in the aftermath of conflict [22,43]. These possible adverse implications also need to be considered while developing initiatives to foster reconciliation and mutual understanding.

Another key finding of the present study was that the number of event types as well as physical health and social integration explained the biggest part of the variance of posttraumatic stress symptoms in the parent generation. The presently observed dose-effect of the number of traumatic event types on the PTSD symptom severity score – highlighting the impact of cumulative stress on mental health – has already been widely discussed [44,45]. The relationship between PTSD and lower self-reported physical health and other health problems has also been reported in previous studies [46,47]. In a recently published study by Schaal et al. [7], both physical illness and trauma exposure were the two main predictors for PTSD symptom severity in Rwandan widows and orphans, while social factors were not further differentiated. The authors discussed this association as a possible difficulty of survivors with PTSD for developing effective coping mechanisms to deal with somatic and chronic health problems or, conversely, that the latter might affect them in such a way that they are no longer able to take care of themselves. Our findings demonstrated chronic pain to be the main physical complaint. With regard to the hypothesis that both syndromes mutually maintain each other as, for instance, acute pain proves to be mediated by symptoms of arousal and vice versa [48,49], this offers an alternative explanation of why PTSD continues to occur at this level, even 16 years after the genocide. Recovery without any treatment or only basic medical-oriented services seem to be reserved to only a fraction of the population. While no evidence for direct prediction of PTSD symptom severity using economic factors was found, physical illness seems to function as a mediator between both, as its correlates with social as well as economic factors demonstrate. The direct consequences of genocidal violence such as HIV infection, chronic pain or disability, which were especially present in the group of survivors, have an immediate impact on the economic growth of a family in an already poor environment [50]. The respondents mainly worked as peasants, as is common for the rural Rwandan population. Therefore, as reported by an interviewed female survivor, when a widow was a victim of sexual violence during the genocide and, due to continued bleeding and other associated physical ailments, was no longer able to perform hard farm labor, her family’s economic status was subsequently and negatively impacted. Even if this kind of survivor were willing to receive psychological support, the challenges of the distance from home to center, money for travel and privacy from neighbors highlight the delicate interconnectedness of economic and social issues in this region.

The present study revealed that lower levels of social integration and activity were associated with elevated levels of PTSD symptoms, indicating a mutual maintenance effect. Interestingly, women in our sample had a lower social status than men, whereas no difference was found either between survivors and former prisoners or between widowed and non-widowed persons. One possible explanation for these results might be that especially vulnerable groups such as widows or genocide survivors infected with HIV tend to stick together and support each other, which is not necessarily the case with people currently suffering from PTSD [51]. According to our findings, genocide survivors had lost an average of 14 family members. In Rwanda, family and community support is crucial for the well-being as well as for the reputation and prosperity of individuals. Depressive moods and feelings of hopelessness in survivors, therefore, were often linked to this missing support, as reported by respondents. As remarriage is socially not necessarily tolerated, widows have difficulties taking care of their family in an appropriate way, especially when they are not further integrated in the community [52].

In comparison to this, former prisoners showed a better economic profile, but surprisingly no higher level of social activity and integration. In our study, while released prisoners rarely mentioned problems of reintegration following their release, their other family members such as their wives and children did. As Richters et al. [50] demonstrated, sources for ongoing conflicts might be found within families in which the father is extensively absent due to imprisonment. In such circumstances, the man returning home from prison may find that his wife has brought another man home or has even had children with other men. Additionally, there has been sharp economic decline and property loss – all factors leading to the father’s realization that he has limited authority in the new family system facing him following imprisonment [53]. Alcohol abuse and the aggressive behavior of released prisoners toward family members or, on the contrary, social withdrawal might also affect family dynamics. As several researchers have demonstrated, mistrust in the communities started to grow again after the first massive release of prisoners in 2003 in Rwanda, many of whom have since been presumed innocent or assigned to minor offenses. Alliances that had previously been made between, for example, genocide widows and wives of prisoners, were thereby once again put into question [54,55]. A large portion of our sample of former prisoners was released within about the same period of time and therefore took part “in the government confession program” that provided a reduced sentence for perpetrators who admitted guilt and remorse [56,57]. Released prisoners expressed being especially grateful to the government as well as for the introduction of the *Gacaca* tribunals, as most of them were released due to this new judicial initiative. At the same time, the majority felt that their own suffering due to imprisonment did not receive any recognition. Therefore, one can argue that social reintegration as a so-called aim of governmental-driven directions on how to behave when going back to their families and communities [56,58] might not necessarily be experienced by the individual former prisoner. This also includes the notion of being innocent and a victim of “someone else’s war” [59], as, in the present study, released prisoners frequently reported physical attack or incarceration as their worst experiences according to the PSS-I. These results are in line with data from a recently published study on incarcerated, accused perpetrators in Rwandan prisons [9]. Only 13% reported their participation in murder as their most stressful event; Schaal et al. thus argue whether high rates of mental health problems in this population are in fact due to the causes or the consequences of imprisonment.

These results highlight the ambiguous and complex nature of victimhood in post-conflict societies as well as the need for further empirical evidence and lead to a first limitation of the study: comparing survivors with former prisoners seems critical with regard to its restriction in conceptualization. As emphasized by numerous authors, dichotomous categories are inappropriate when describing and reflecting complex circumstances of life in and after wartimes [12]. A dynamic view on participation is required whenever a better understanding of the role and participation in periods of violence is in question. People do not often fit to one category alone, such as victim, perpetrator or bystander, and can change throughout time in their concrete behavior. This change and assumption of different positions within the same period of time could, therefore, influence the impact on their mental health situation. Following Bar-On [60] there might be an association between the use of interchangeable roles and reduced moral responsibility in a person, which together could have a disburdening effect. Therefore, one individual who protected and rescued a nearby Tutsi neighbor (who in turn survived the genocide) while having also participated in roadblocks and manhunts for the Tutsi might be a conceivable example of how one person could incorporate many roles at the same time. Our findings might not necessarily hold for the whole Rwandan population. As already demonstrated, results from different regions might vary and our data can therefore only be seen as representative for central Gitarama. Still, they offer initial insight into a local population consisting of families of survivors and former prisoners living next door to each other who had never before been examined together and compared with each other. Another limitation lies in our definition of family. In Rwandan families, older children in particular do not necessarily grow up near their parents or siblings, but often refer to the broader family context including uncles, aunts, or even grandparents as their parents or to cousins as their siblings as well. The present study does not provide further information about the previous childhood circumstances of descendants in order to draw further conclusions on potential protective factors in the aftermath of violence. Finally, conducting research in a post-conflict setting demanded a critical reflection upon data validity. Due to political restriction and oppression, speaking out loudly is not common in Rwanda. The effect of introducing local interviewers also needs to be taken into consideration. While local researchers normally benefit from their close relationships to the cultural background in question, the specific historically shaped relationships between Rwandans might sometimes foster an even stronger mistrust between Rwandans than toward foreigners.

## Conclusion

16 years following the Rwandan genocide, survivors and their families continue to present with considerable rates of PTSD and substantial depressive and anxiety symptoms. The data revealed a strong association between health problems and psychosocial factors such as social integration. A high level of PTSD in the group of former prisoners – and thus the likely genocide perpetrators – demonstrated that psychological suffering affected the population at large, although the nature of traumatic stressors may have varied. By challenging the question of who “owns” trauma discourses in a post-conflict society such as Rwanda, mental health services should take the needs of the entire population into consideration when offering care – an idea that is still not mainstream in Rwanda. Finally, descendants of genocide survivors showed a higher risk for mental health problems than descendants of former prisoners. A high trauma load as well as missing family integration and support characterizes their specific vulnerable situation. Interventions and initiatives that stimulate reconciliation processes between future generations should take these particularities into consideration.

## Competing interests

The authors declare that they have no competing interests.

## Authors’ contributions

HR conceived and designed the study and coordinated and supervised data acquisition. She was responsible for statistical analysis and drafted the manuscript. TE participated in the study design, the analysis of the data and manuscript preparation. All authors read and approved the final manuscript.
